# Real world data of cabozantinib in patients with hepatocellular carcinoma: Focusing on dose setting and modification

**DOI:** 10.1002/cam4.70222

**Published:** 2024-09-24

**Authors:** Hironao Okubo, Hitoshi Ando, Shunsuke Nakamura, Yusuke Takasaki, Koichi Ito, Yuka Fukuo, Kenichi Ikejima, Hiroyuki Isayama

**Affiliations:** ^1^ Department of Gastroenterology Juntendo University Nerima Hospital Tokyo Japan; ^2^ Department of Gastroenterology Juntendo University School of Medicine Tokyo Japan; ^3^ Department of Cellular and Molecular Function Analysis Kanazawa University Graduate School of Medical Sciences Ishikawa Japan

**Keywords:** aldolase, cabozantinib, dose modification, hepatocellular carcinoma, muscle injury, relative dose intensity

## Abstract

**Aim:**

To investigate the outcomes of cabozantinib in patients with unresectable hepatocellular carcinoma (uHCC), focusing on dose setting and modification.

**Methods:**

We retrospectively analyzed 34 Japanese patients who received cabozantinib for uHCC. Trough concentrations (C_trough_) of cabozantinib were also measured weekly for 6 weeks in the 18 patients.

**Results:**

Sixteen patients received ≥40 mg (high‐dose group), and 18 patients received 20 mg (low‐dose group). Dose escalations were performed in 27.8% of the patients in the low‐dose group. Although median duration of the first dose reduction or interruption in the low‐dose group was twice that in the high‐dose group (28 vs. 14 days, *p* < 0.001), there were no significant differences in the relative dose intensity (RDI) during 6 weeks, progression free survival (PFS), and overall survival (*p* = 0.162, *p* = 0.950, *p* = 0.817, respectively) between the two groups. Patients who received RDI during 6 weeks ≥33.4% showed a trend toward longer median PFS (*p* = 0.054). Each serum aldolase value during the 6 weeks was significantly correlated with the C_trough_ at any point (*r* = 0.500, *p* < 0.001). In multivariate analyses, aldolase ≥8.7 U/L within 2 weeks was significantly associated with the very early dose reduction or interruption (odds ratio 20.0, *p* = 0.002).

**Conclusions:**

An initial dose of 20 mg cabozantinib could be a safe option in Japanese patients. The serum aldolase value could be useful for making appropriate dose modifications of cabozantinib.

## 1 INTRODUCTION

Hepatocellular carcinoma (HCC) is the fifth most common cancer worldwide.^1^ Cabozantinib is an oral multi tyrosine kinase inhibitor (TKI) that inhibits vascular endothelial growth factor receptor 1–3, growth arrest‐specific 6 receptor, and hepatocyte growth factor receptor.^2^ Cabozantinib is a candidate for patients with unresectable HCC (uHCC) following the use of other systemic therapies.^3^ Cabozantinib has also been utilized for patients with renal cell carcinoma, advanced differentiated thyroid cancer, and metastatic medullary thyroid cancer.

In the phase 3 CELESTIAL trial for patients with uHCC previously treated with sorafenib, those with cabozantinib treatment had a significant survival benefit compared with those with the placebo.^3^ Although the initial dose of cabozantinib for HCC was 60 mg once daily according to the product document, dose reduction and interruption due to several treatment‐related adverse events (AEs) occurred in 62 and 84% of patients in the CELESTIAL trial, respectively, and 91 and 91% of patients in the Japanese phase 2 trial, respectively.^3^, ^4^

Recent real‐world data on cabozantinib for uHCC showed that reduced starting doses of 20 or 40 mg once daily were used in 4.4%–28% and 31%–63% of cases, respectively.^5^, ^6^, ^7^, ^8^ This suggests that setting the initial dose and adjusting it during treatment can be challenging in clinical practice. Consequently, adequate dose setting and careful dose modification are required to maintain the quality of cabozantinib pharmacotherapy for uHCC. Adjusting cabozantinib dosage by plasma level monitoring may facilitate management for patients who develop intolerable AEs. The examination of drug concentration is unrealistic in the clinical settings, therefore, elucidation of a standard value reflecting drug exposure of cabozantinib is urgently needed. Furthermore, whether reduced starting dose of cabozantinib, such as 20 mg, would be feasible regarding efficacy requires verification. Nevertheless, the relationship between the efficacy and safety of a starting dose of 20 mg and those of 40 mg or more has not been well established for patients with HCC treated with cabozantinib. Therefore, the aim of this study was to analyze clinical efficacy and safety in patients with uHCC based on the starting dose of cabozantinib. The secondary aim was to find surrogate markers useful in AE management of cabozantinib.

## 2 MATERIALS AND METHODS

### 2.1 Patients

In this two‐facility retrospective observational study, we enrolled 34 consecutive Japanese patients with uHCC who were treated with cabozantinib after the progression of other systemic therapies between March 2021 and December 2023 at the Juntendo University Nerima Hospital (*n* = 25) and Juntendo University School of Medicine (*n* = 9). The present study included the patients from the cohort of our previous study.^9^ The study was approved by the Ethical Committee of our university (Approval No. E23‐0193) and was performed in accordance with the ethical standards of the 1964 Declaration of Helsinki and its later amendments. All informed consent was obtained from all the patients to participate in the study. Patients were administered oral cabozantinib (CABOMETYX; Exelixis, Inc. Alameda, CA, USA) at 60, 40, or 20 mg once daily as the initial dose, according to the decision of the attending doctors. The patients visited our institution 7, 14, 21, 28, and 42 days after starting treatment, and blood samples were collected at every visit. AEs were evaluated according to the Common Terminology Criteria for Adverse Events (CTCAE) version 5.0. According to the guide for cabozantinib administration, dose reductions (60–40 mg, 40–20 mg, 20 to alternate‐day 20 mg) or dose interruption were allowed in cases of any CTCAE grade 3 or 4 or any unacceptable CTCAE grade 2. If tolerated, dose escalations of cabozantinib were considered. The therapeutic response was assessed by enhanced computed tomography that was pictured 6 weeks after starting treatment and every 8–12 weeks after that, in accordance with the Response Evaluation Criteria in Solid Tumors (RECIST) version 1.1. The RDI was defined as the actual cumulative administered dose during 2, 4, and 6 weeks by that of 60 mg once daily for the same duration.

### 2.2 Cabozantinib pharmacokinetic analysis

Plasma samples of trough values in cabozantinib were collected on day 7, 14, 21, 28, and 42 for the 18 patients. The samples were isolated through centrifugation within 30 min at 1500 rpm and 4°C for 10 min. Plasma cabozantinib concentrations (C_trough_) were then measured using liquid chromatography–tandem mass spectrometry, as described.^9^

### 2.3 Statistical analysis

Continuous variables are shown as median values (range) or mean ± standard deviation and examined using the Mann–Whitney *U*‐test or Student *t*‐test. Differences in serum parameters between three groups were analyzed using the Kruskal–Wallis test. When significant differences were observed in the Kruskal–Wallis test, corrections for multiple pairwise comparisons were made according to Bonferroni's method.

Each categorical data was also tested using chi‐square or Fisher's exact test. Differences in longitudinal data of relative dose intensity (RDI) between the high and low‐dose group were tested by repeated measures using ANOVA. Therapeutic outcomes, such as progression free survival (PFS) and overall survival (OS), were established using the Kaplan–Meier method. Differences in the two groups were examined using the log‐rank test. Spearman's correlation coefficient was used to determine the relationships between pairs of variables. Variables of factors related to the very early dose reduction or interruption of cabozantinib with *p* < 0.05 in the univariate analysis were reevaluated by multiple logistic regression analysis using the forward selection method. All tests were two‐sided, and statistical significances were set at *p* < 0.05. All statistical analyses were performed using SPSS Statistics for Windows, Version 27 (IBM Corp., Tokyo, Japan).

## 3 RESULTS

### 3.1 Baseline characteristics

The baseline characteristics of all Japanese patients are shown in Table 1. Median duration of observation from cabozantinib initiation was 8.5 months (interquartile range, 3.6–14.0 months). Median age was 74 years (range, 29–91 years). Twenty‐eight patients (82.4%) were men. Median body weight was 57.4 kg (range, 39–88.2 kg). Treatment lines consisted of 10 in second, 20 in third, 2 in fourth, and 2 in fifth. Four patients received 60 mg, 12 patients received 40 mg, and 18 patients received 20 mg of cabozantinib as the initial dose. Twenty‐eight patients (82.4%) had Child–Pugh class A.

**TABLE 1:** Patient characteristics in all patients and comparison of those treated with cabozantinib as high starting dose (≥40 mg) (high‐dose group) and low starting dose (20 mg) (low‐dose group).

	Total	High‐dose group	Low‐dose group	*p‐*value
	*N* = 34	*n* = 16	*n* = 18	
Age, year	74 (29–91)	78 (56–87)	72 (29–91)	0.175
Sex, male/female	26/8	14/2	12/6	0.232
Performance status ECOG: 0/1	28/6	12/4	16/2	0.387
Etiology: HBV/HCV/non‐viral	4/7/23	1/3/12	3/4/11	0.882
Body weight, kg	57.4 (39–88.2)	60 (39–88.2)	55.1 (44–70)	0.443
Body mass index	22.5 (14.8–31.6)	22.8 (14.8–31.6)	21.3 (18.1–26.6)	0.874
Body surface area, m^2^	1.63 (1.32–1.97)	1.70 (1.37–1.97)	1.57 (1.32–1.80)	0.158
BCLC stage: B/C	13/21	7/9	6/12	0.725
Intrahepatic tumor number, none/1–5/≥6	6/6/22	1/2/13	5/4/9	0.353
Maximus intrahepatic tumor diameter, cm; none/≤5/>5	6/18/10	1/10/5	5/8/5	0.490
Extrahepatic spread: Yes/No	18/16	7/9	11/7	0.504
Macro vascular invasion: Yes/No	6/28	4/12	2/16	0.378
Treatment line: 2nd/3rd/4th/5th	10/20/2/2	6/8/1/1	4/12/0/1	0.551
Starting dose: 60/40/20 mg	4/12/18	4/12/0	0/0/18	<0.001
Past history of ATZ + BEV: Yes/No	23/11	8/8	15/3	0.0662
Past history of lenvatinib: Yes/No	33/1	16/0	17/1	1.000
Child‐Pugh score: 5/6/7/8	17/11/4/2	9/4/3/0	8/7/1/2	0.801
mALBI grade: 1/2a/2b	6/14/14	3/7/6	3/7/8	0.963
Total bilirubin, mg/dL	0.8 (0.4–2.4)	0.8 (0.4–1.6)	0.5 (0.5–2.4)	0.07
Albumin, g/dL	3.6 (3.0–3.4)	3.6 (3.0–4.4)	3.5 (3.1–4.4)	0.772
Aspartate aminotransferase, U/L	48 (19–157)	51 (22–157)	44 (19–68)	1.000
Alanine aminotransferase, U/L	28 (11–115)	24 (11–115)	32 (14–69)	0.384
Lactic acid dehydrogenase, U/L	223 (149–329)	228 (150–329)	218 (149–296)	0.851
Prothrombin activity, %	89 (62–116)	94 (62–116)	88 (66–114)	0.746
eGFR, mL/dL/1.13m^2^	63.77 (23.39–110.8)	67.89 (23.29–110.8)	61.14 (40.93–94.21)	0.330
Creatine kinase, U/L	77 (28–189)	72 (28–162)	92 (29–189)	0.574
Aldolase, U/L	5.2 (3.1–7.6)	5.2 (3.1–6.1)	4.3 (3.3–7.6)	0.259
AFP, ng/mL	43.5 (1.2–178,562)	213.0 (1.4–178,562)	14 (1.2–14,293)	0.033
DCP, mAU/mL	750 (20–78,100)	3260 (20–78,100)	265 (27–16,400)	0.059

*Note*: Median (range) or *n*.

Abbreviations: AFP, ɑ‐fetoprotein; ATZ, atezolizumab; BCLC, Barcelona Clinic Liver Cancer; BEV, bevacizumab; DCP, des‐carboxy prothrombin; eGFR, estimated glomerular filtration rate; HBV, hepatitis B virus; HCV, hepatitis C virus; mALBI, modified albumin‐bilirubin; PS, performance status.

### 3.2 Comparison of baseline factors in patients with cabozantinib as high starting dose and low starting dose

Based on the initial dose of cabozantinib, the patients were classified into two groups. A comparison of patients treated with cabozantinib as a high starting dose (≥40 mg) (high dose group) and a low starting dose (20 mg) (low‐dose group) is also listed in Table 1. Specifically, 16 patients (47%) received ≥40 mg and 18 patients (53%) received 20 mg. There were no significant baseline factors between the two groups excluding ɑ‐fetoprotein (AFP) value.

### 3.3 Therapeutic details of cabozantinib

Therapeutic details of the low and high dose groups are shown in Table 2. The median duration of the first dose reduction or interruption in the high dose group was significantly shorter that in the low‐dose group (14 vs. 28 days, *p* < 0.001). During the first 6 week of cabozantinib treatment, 89.1% in all patients experienced dose reduction or interruption due to AEs. Notably, the rate of dose reduction or interruption rate in the high dose group was 43.7% and 100% at 1 and 6 weeks, respectively. In contrast, the rate of dose reduction or interruption rate in the low‐dose group during at 6 weeks was 79.7%. The median duration of the first dose interruption in the high dose group was significantly shorter than in the low‐dose group (7 vs. 119 days, *p* = 0.012). Median duration of the first interruption in the all patients was 28.0 days (95% CI 4.4–51.6). The dose interruption rate at 6 weeks in the high‐dose group were 81.2%, while that in the low‐dose group was 36.8%. There was no significant difference in the average daily dose in the first 6 weeks between the high‐ and low‐dose groups (20 vs. 19.5 mg, *p* = 0.350). The dose escalation rate during the 6 weeks was 0% (0/16) in the high‐dose group, in contrast, it was 27.8% (5/18) in the low‐dose group. There was no significant difference in the total duration of drug exposure between the high and low‐dose groups (70 vs. 120 days, *p* = 0.882).

**TABLE 2:** Therapeutic details of cabozantinib treatment.

	Total	High‐dose group	Low‐dose group	*p*‐value
	*N* = 34	*n* = 16	*n* = 18	
Starting dose, 60/40/20 mg	4/12/18	4/12/0	0/0/18	<0.001
Median duration of the first dose reduction or interruption, days (95% CI)	14 (8.9–19.1)	14 (9.8–18.2)	28 (21.1–34.9)	<0.001
Rate of dose reduction or interruption rate during at 1 week, %	20.6	43.7	0	
Rate of dose reduction or interruption rate during at 2 weeks, %	51.1	78.9	27.8	
Rate of dose reduction or interruption rate during at 4 weeks, %	81.9	93	72.9	
Rate of dose reduction or interruption rate during at 6 weeks, %	89.1	100	79.7	
Median duration of the fist dose interruption, days (95% CI)	28 (4.4–51.6)	7 (NE–NE)	119 (20.5–217.6)	0.012
Rate of dose interruption rate during at 1 week, %	23.5	50	0	
Rate of dose interruption rate during at 2 weeks, %	35.3	68.7	5.6	
Rate of dose interruption rate during at 4 weeks, %	51.1	81.2	24.1	
Rate of dose interruption rate during at 6 weeks, %	57.6	81.2	36.8	
Median average daily dose during 6 weeks, mg (95% CI)	20.0 (17.4–23.2)	20.0 (17.2–27.6)	19.5 (15.4–21.5)	0.350
Dose escalation rate during 6 weeks, %	14.7	0	27.8	0.046
Median duration of drug exposure, days (95% CI)	105 (24.7–185.3)	70 (42.2–97.8)	120 (93.9–146.1)	0.913

Abbreviations: CI, confidence interval; NE, not evaluable.

### 3.4 Time course changes in RDI between the high‐ and low‐dose group

**FIGURE 1:**
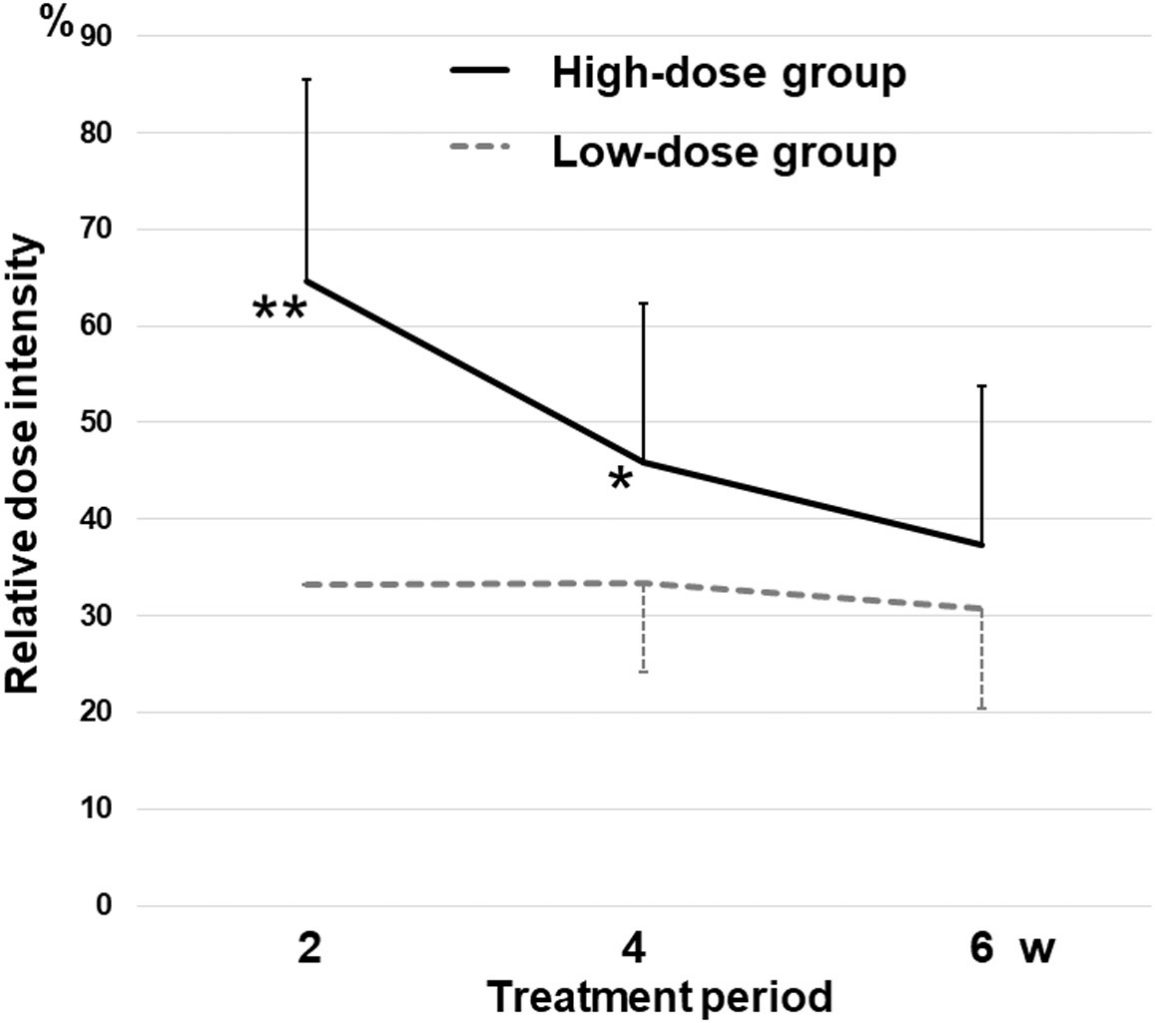
Time course changes in relative dose intensity of cabozantinib between the high‐dose (≥40 mg) group and the low‐dose (20 mg) group as an initial dose. **p < 0.001 versus low‐dose group, *p < 0.05 versus low‐dose group.

Time course changes in RDI of cabozantinib at 2, 4, and 6 weeks are shown in Figure 1. Significant differences in the RDI in the high‐dose group among 2, 4, and 6 weeks were found (*p* < 0.001), while the values in the low‐dose group among 2, 4, 6 weeks were not significantly different (*p* = 0.305). The RDI in the high‐dose group was significantly higher than that in the low‐dose group at 2 and 4 weeks (64.6 ± 21.0% vs. 33.3 ± 0%, *p* < 0.001; 45.9 ± 16.4% vs. 33.5 ± 9.2%, *p* = 0.013, respectively). There was no significant difference in the RDI at 6 weeks between the high‐ and low‐dose group (37.34 ± 16.39% vs. 30.7 ± 10.28%, *p* = 0.162).

### 3.5 Therapeutic outcome

Therapeutic effects, such as the best antitumor response are listed in Table 3. The overall best objective response rate (ORR) and disease control rate (DCR) were 5.9% and 67.6%, respectively. The best ORR and DCR were 0% and 68.8% in the high‐dose group and 11.1% and 66.7% in the low‐dose group, respectively. There were no significant differences in the ORR and DCR between the two groups (*p* = 0.487 and *p* = 0.897, respectively).

**TABLE 3:** Treatment effect.

	Total	High‐dose group	Low‐dose group
	*N* = 34	*n* = 16	*n* = 18
Best overall response			
CR, *n* (%)	0	0	0
PR, *n* (%)	2 (5.9)	0	2 (11.1)
SD, *n* (%)	21 (61.8)	11 (68.8)	10 (55.6)
PD, *n* (%)	8 (23.5)	3 (18.8)	5 (27.8)
NE, *n* (%)	3 (8.8)	2 (12.5)	1 (5.6)
ORR, %	5.9	0	11.1
DCR, %	67.6	68.8	66.7

Abbreviations: CR, complete response; DCR, disease control rate; NE, not evaluable; ORR, objective response; PD, progressive disease; PR, partial response; SD, stable disease.

**FIGURE 2:**
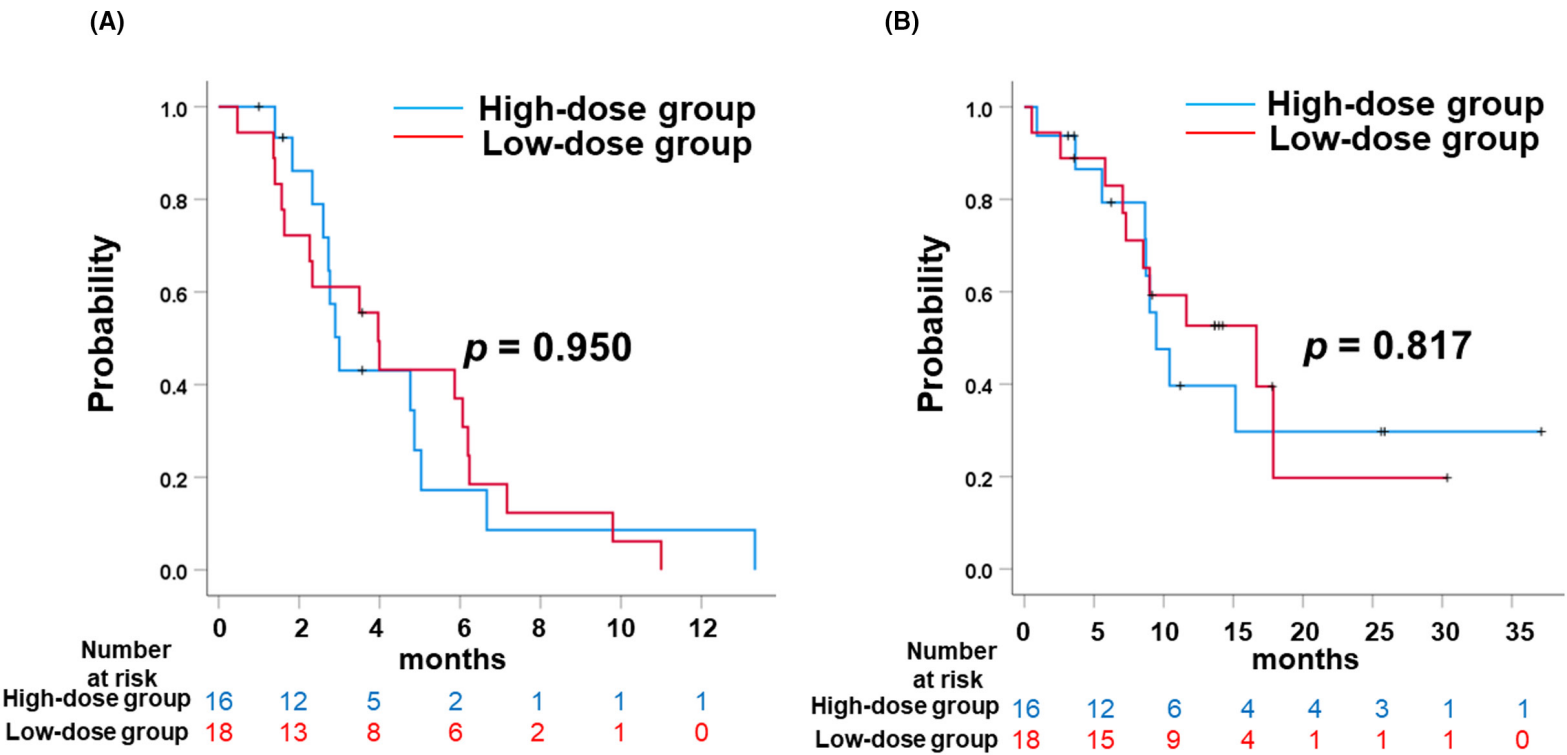
Comparison of progression free survival (A), and overall survival (B) between the high dose (≥40 mg) group and the low dose (20 mg) group as an initial dose of cabozantinib.

Kaplan–Meier curves for PFS and OS in patients stratified by the starting dose are shown in Figure 2. The median PFS of the patients in high‐ and low‐dose groups were 3.0 months (95% confidence interval (CI): 2.58–3.42) and 4.0 months (95% CI: 3.02–4.91), respectively. The median OS of the patients in high‐ and low‐dose groups were 9.5 months (95% CI: 7.0–11.9) and 16.7 months (95% CI: 5.5–27.8), respectively. No significant differences were found in the PFS and OS between the two groups (*p* = 0.950 and *p* = 0.817, respectively). The PFS and OS for all patients were 4.0 months (95% CI: 2.5–5.4 months) and 11.6 months (95% CI: 5.6–18.3 months), respectively.

**FIGURE 3:**
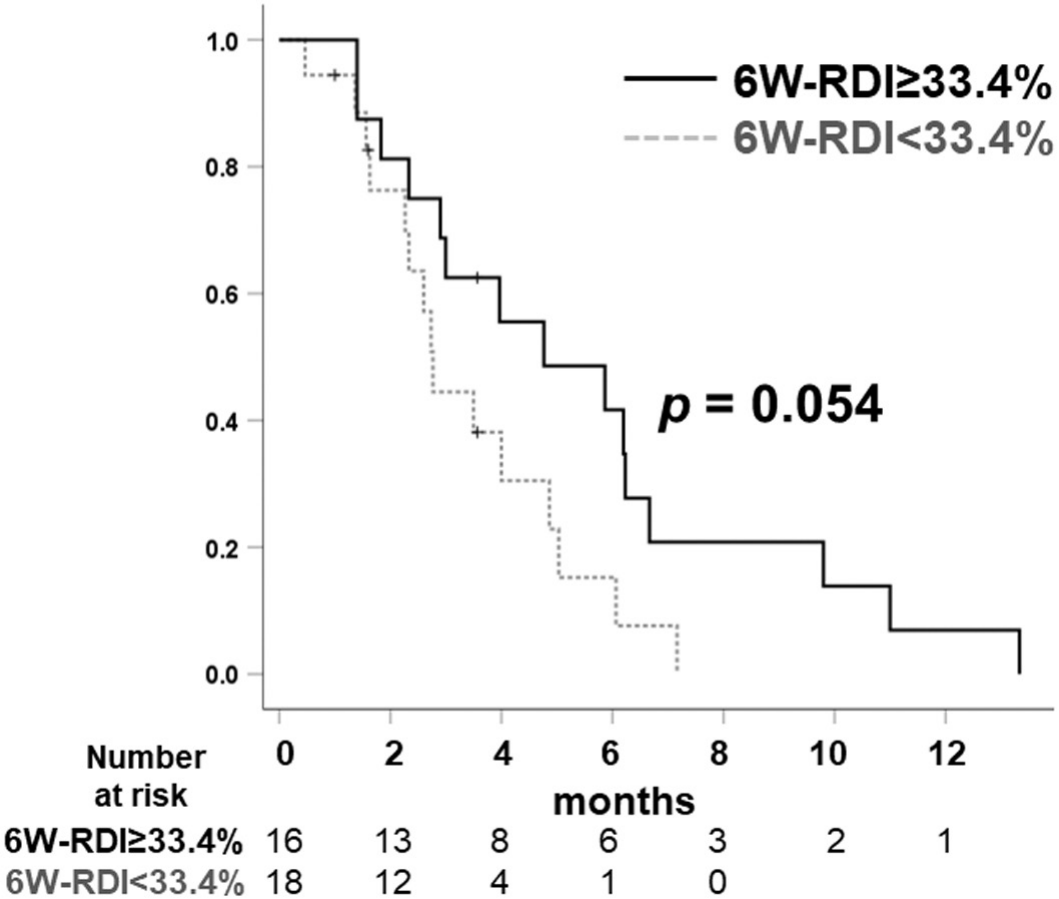
Comparison of progression free survival of patients treated with cabozantinib between the group of relative dose intensity during 6 weeks (6 W‐RDI) ≥33.4 mg and those of 6 W‐RDI<33.4 mg.

As the median RDI during 6 weeks (6 W‐RDI) in all patients was 33.3%, the relationships of PFS between the 6 W‐RDI≥33.4% and 6 W‐RDI<33.4% are shown in Figure 3. The 6 W‐RDI≥33.4% group comprised 56.3% (9/16) in the high‐dose group and 38.9% (7/18) in the low‐dose group, the proportion of which was not significant (*p* = 0.504). Patients who received 6 W‐RDI≥33.4% of cabozantinib showed a trend toward longer median PFS (4.8 months; 95% CI: 1.4–8.2) than the other patients (2.8 months; 95% CI: 2.4–3.1), although the difference was not statistically significant (*p* = 0.054).

### 3.6 Adverse events

AEs within 6 weeks after the start of cabozantinib treatment according to the high‐ and low‐dose groups are listed in Table 4. AEs of any grade and grade 3 or more occurred in 100% and 47.2% of the patients in our cohort, respectively. Dose reduction or discontinuation was not only attributed to the occurrence of grade 3 AEs but also to non‐acceptable grade 2 AEs. Specifically, eight of 16 patients (50%) in the high‐dose group and six of 18 patients (33.3%) in the low‐dose group experienced drug reduction or discontinuation due to unacceptable grade 2 AEs.

**TABLE 4:** Adverse events.

	Any grade				Grade 3 or more			
	All, *N* = 34	High‐dose group, *n* = 16	Low‐dose group, *n* = 18	*p*‐value	All, *N* = 34	High‐dose group, *n* = 16	Low‐dose group, *n* = 18	*p*‐value
	*n*, (%)	*n*, (%)	*n*, (%)		*n*, (%)	*n*, (%)	*n*, (%)	
Any adverse event	34 (100)	16 (100)	18 (100)	1.00	16 (47.1)	8 (50.0)	8 (44.4)	0.984
Malaise	20 (58.8)	11 (68.8)	9 (50.0)	0.447	7 (20.6)	5 (31.3)	2 (11.1)	0.214
Proteinuria	18 (52.9)	11 (68.8)	7 (38.9)	0.162	4 (11.8)	3 (18.8)	1 (5.6)	0.323
Hand and foot syndrome	13 (38.2)	4 (25.0)	9 (50.0)	0.252	1 (2.9)	0	1 (5.6)	1.00
Anorexia	12 (35.3)	9 (56.3)	3 (16.7)	0.030	2 (5.9)	1 (6.3)	1 (5.6)	1.00
Hypothyroidism	9 (26.5)	5 (31.3)	4 (22.2)	0.837	0	0	0	1.00
Diarrhea	8 (23.5)	5 (31.3)	3 (16.7)	0.429	3 (8.8)	2 (12.5)	1 (5.6)	0.591
Hypertension	12 (35.3)	7 (43.8)	5 (27.8)	0.540	0	0	0	1.00
Increased AST	19 (55.9)	12 (75.0)	7 (38.9)	0.077	1 (2.9)	1 (6.3)	0	1.00
Increased ALT	20 (58.8)	11 (68.8)	9 (50.0)	0.447	0	0	0	1.00
Increased CK	9 (26.5)	6 (37.5)	3 (16.7)	0.250	0	0	0	1.00

Abbreviatons: ALT, alanine aminotransferase; AST, aspartate aminotransferase; CK, creatine kinase.

Malaise, which was the most frequent AE for dose reduction or interruption, was observed in 58.8% of the patients in any grade including 20.6% in grade 3 or more. Other frequent AEs in any grade were increased alanine aminotransferase in 58.8%, increased aspartate aminotransferase in 55.9%, and proteinuria in 52.9% of all patients. Anorexia occurred in 12 of all 34 patients (35.3%), grade 1 in 6, grade 2 in 4, and grade 3 or more in 2. Notably, increased creatine kinase (CK) was occurred in 26.5% of all 34 patients without muscle weakness and muscle pain.

The high‐dose group developed any grade of anorexia significantly higher than low‐dose group (56.3 vs. 16.7%, *p* = 0.030). Additionally, the high‐dose group tends to develop increased aspartate aminotransferase compared with that in the low‐dose group (75.0 vs. 38.9%, *p* = 0.077). There were no significant differences in the developing rate regard AEs of grade 3 or more between the two groups.

### 3.7 Changes in serum parameter and correlation between serum parameters and cabozantinib exposure

The time course changes in serum aldolase, CK, aspartate aminotransferase, alanine aminotransferase, and lactic acid dehydrogenase (LDH) levels in all 34 patients are illustrated in Figure S1. The values at 1, 2, 3, 4, and 6 weeks were significantly higher than those at the baseline values. In particular, all patients exhibited serum aldolase values exceeding the standard level throughout the initial six‐week treatment period.

**FIGURE 4:**
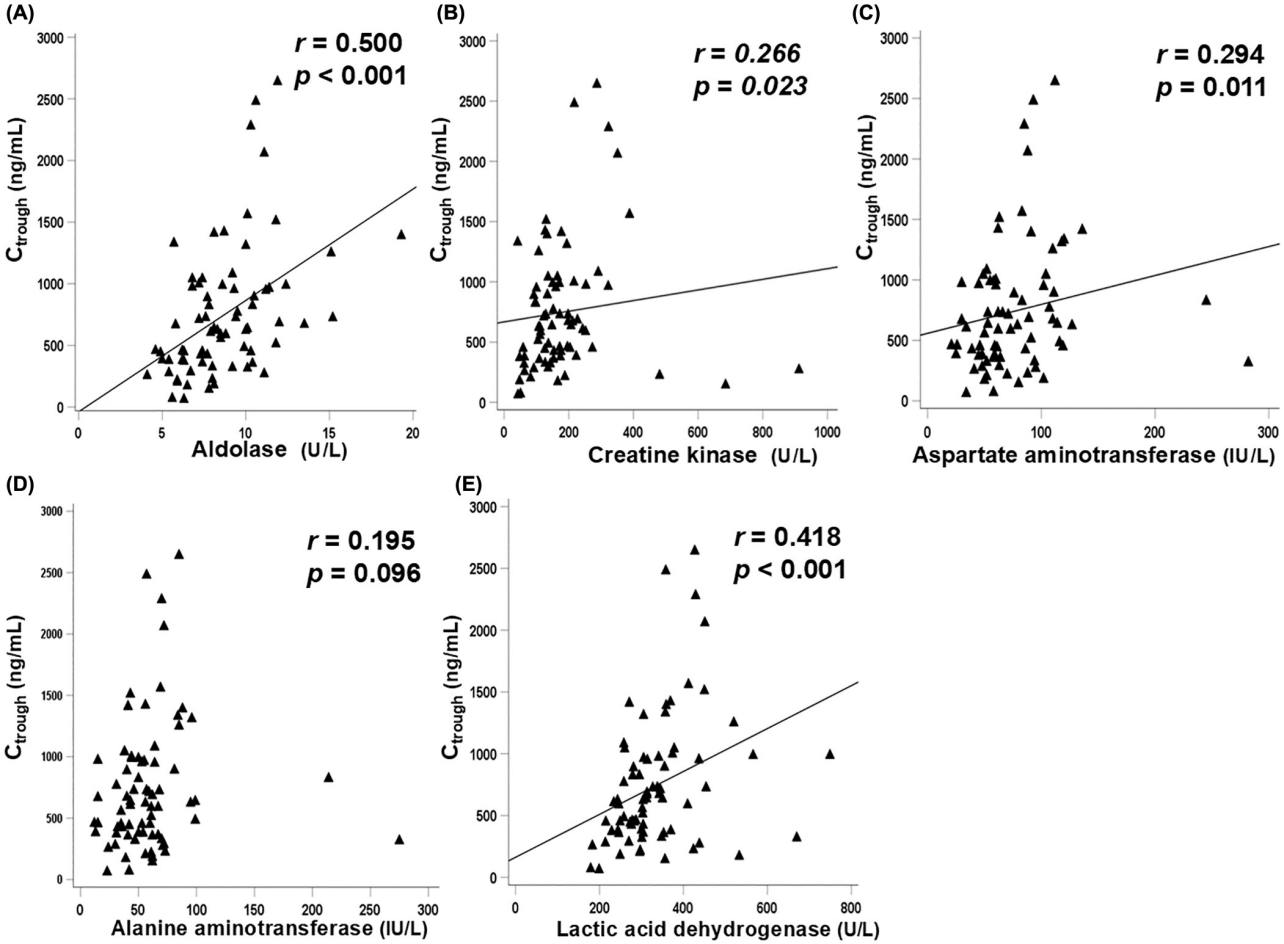
Correlations between all values of aldolase (A), creatine kinase (B), aspartate aminotransferase (C), alanine aminotransferase(D), and lactic acid dehydrogenase (E) and trough concentration (C_trough_) of cabozantinib at any point.

Associations between the serum values of aldolase, CK, aspartate aminotransferase, alanine aminotransferase, and LDH and the C_trough_ of cabozantinib at the same point during the 6 weeks in 18 patients are presented in Figure 4. There were significant positive correlations between all values of aldolase, CK, aspartate aminotransferase, and LDH and the C_trough_ of cabozantinib (*r* = 0.500, *p* < 0.001; *r* = 0.266, *p* = 0.023; *r* = 0.294, *p* = 0.011; and *r* = 0.418, *p* < 0.001, respectively) (Figure 4A–D). In contrast, there was no significant correlation between all values of alanine aminotransferase and the C_trough_ of cabozantinib (*r* = 0.195, *p* = 0.096) (Figure 4D).

### 3.8 Relationship between anorexia and serum parameters

**FIGURE 5:**
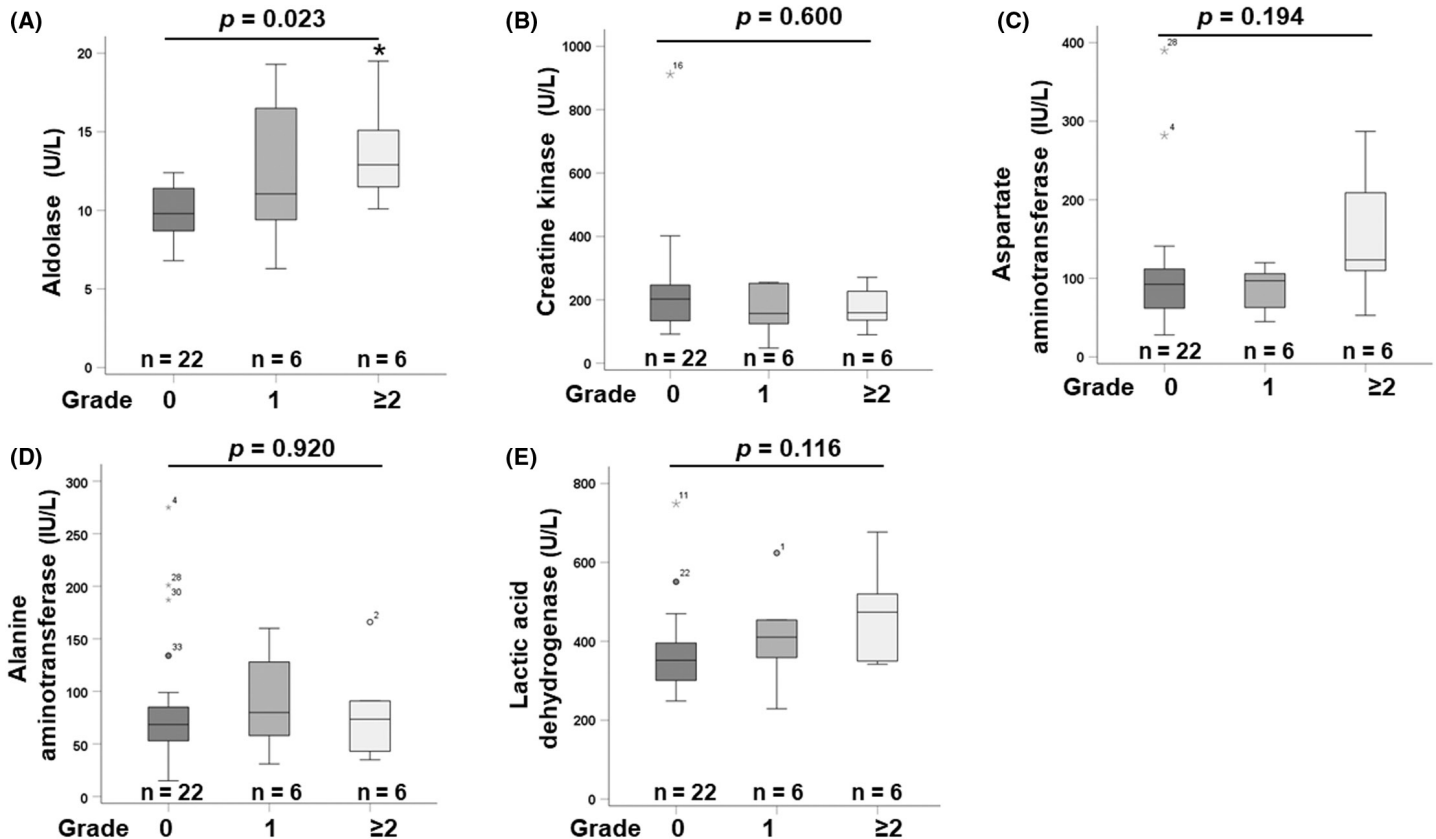
Relationship between CTCAE grade of anorexia and serum maximum levels of aldolase (A), creatine kinase (B), aspartate aminotransferase (C), alanine aminotransferase(D), and lactic acid dehydrogenase (E) using a box and whisker plot. The box spans data between the two quartiles (interquartile range [IQR]), and the horizontal line in the center of each box represents the median of each group. The ends of the whiskers represent the largest and smallest values that are not outliers. The outliers are value between 1.5 and three IQRs from the end of the box. *p < 0.05, versus grade 0 group.

Relationships between CTCAE grade of anorexia (grade 0, grade 1, and grade 2 or more) and serum parameters are shown in Figure 5. There were significant differences among the three groups in maximum serum aldolase value during 6 weeks (*p* = 0.023) and post‐hoc pairwise comparison test revealed significantly higher aldolase values in grade 2 or more than in those with grade 0 (*p* = 0.021). In contrast, there were no significant differences among the three groups in maximum levels of CK, aspartate aminotransferase, alanine aminotransferase, and LDH (*p* = 0.600, *p* = 0.194, *p* = 0.920, and *p* = 0.166, respectively).

### 3.9 Receiver operating characteristics curve analysis in prediction of onset of cabozantinib dose reduction or interruption

A receiver operating characteristic (ROC) curve analysis was conducted and the area under the ROC curve (AUROC) was calculated to determine the best cutoff levels of each parameter at 1, 2, 3, 4, and 6 weeks for predicting of onset of cabozantinib dose reduction or interruption, which denotes abandonment of the initial dose, during the 6 weeks after starting treatment. As listed in Table 5, ROC analysis indicated that aldolase ≥8.7 U/L, CK≥197 U/L, aspartate aminotransferase ≥111 IU/L, alanine aminotransferase ≥82 IU/L, and LDH ≥342 U/L were the best cutoff values for the dose reduction or interruption of cabozantinib during the 6 weeks. Specifically, serum aldolase ≥8.7 U/L demonstrated a high level of accuracy in predicting dose reduction or interruption, with an AUROC of 0.786.

**TABLE 5:** ROC curve analysis of each parameter and prediction of onset of cabozantinib dose reduction or interruption during 6 weeks.

	Cut off value	Sesitivity, %	Specificity, %	*p*‐value	AUROC (95% CI)	Positive predictive value, %	Negative predictive value, %
Aldolase, U/L	8.7	85.7	78.9	<0.001	0.786 (0.704–0.868)	44.4	95.3
Creatine kinase, U/L	197	42.4	82.4	0.005	0.673 (0.559–0.868)	42.4	85.6
Aspartate aminotransferase, IU/L	111	15.6	87.1	0.194	0.58 (0.467–0.694)	23.8	80.0
Alanine aminotransferase, IU/L	82	28.1	81.5	0.033	0.632 (0.518–0.746)	28.1	81.5
Lactic acid dehydrogenase, U/L	342	71.9	71.8	<0.001	0.716 (0.611–0.821)	39.7	90.8

Abbreviations: AUROC, area under the receiver operating characteristic curve; CI, confidence interval; ROC, receiving operating characteristic.

### 3.10 Factors associated with the very early dose reduction or interruption of cabozantinib

The results of univariate and multivariate logistic regression analyses of factors associated with very early, dose reduction or interruption of cabozantinib within 2 weeks are shown in Table 6. Univariate logistic regression analysis showed that starting dose of cabozantinib ≥40 mg (*p* = 0.026) and serum aldolase value ≥8.7 U/L (*p* = 0.002) were significantly related to very early dose reduction or interruption, whereas a serum aldolase value ≥8.7 U/L was identified as a significant independent factor in multivariate logistic regression analysis (odds ratio 20.0, 95%CI, 3.076–130.059; *p* = 0.002).

**TABLE 6:** Univariate and multivariate analyses of factors related to the very early dose reduction or interruption of cabozantinib.

Variable	Univariate analysis			Multivariate analysis		
	OR	95% CI	*p*‐value	OR	95% CI	*p*‐value
Age, ≥75 years	0.938	0.229–3.385	0.928			
Body weight, <60 kg	0.600	0.152–2.362	0.465			
Sex, male	1.364	0.278–6.683	0.702			
Starting dose, ≥40 mg	7.222	1.268–41.143	0.026	3.354	0.506–22.219	0.210
BCLC stage, C	0.404	0.094–1.733	0.223			
mALBI, 1 or 2a	0.556	0.137–2.256	0.411			
Subsequent administration after lenvatinib	1.571	0.402–6.142	0.516			
eGFR, <60 mL/dL/1.13m^2^	0.313	0.074–1.318	0.113			
1, 2w‐aldolase, ≥8.7 U/L	17.00	2.756–104.54	0.002	20.00	3.076–130.059	0.002
1, 2w‐creatine kinase, ≥197 U/L	4.727	0.825–27.073	0.081			
1, 2w‐aspartate aminotransferase, ≥111 IU/L	3.335	0.780–14.265	0.104			
1, 2w‐alanine aminotransferase, ≥82 IU/L	1.404	0.318–6.160	0.656			
1, 2w‐lactic acid dehydrogenase, ≥342 U/L	1.896	0.467–7.701	0.371			
AFP, ≥129 ng/mL	1.571	0.402–6.142	0.516			
DCP, ≥40 mAU/mL	3.429	0.827–14.209	0.089			

Abbreviations: AFP, ɑ‐fetoprotein; BCLC, Barcelona Clinic Liver Cancer; DCP, des‐carboxy prothrombin, eGFR, estimated glomerular filtration rate; mALBI, modified albumin‐bilirubin; PS, performance status.

## 4 DISCUSSION

In this study, we have shown real‐world data of cabozantinib for uHCC, specifically focusing on dose setting and modification. We found that the high‐dose group required dose reduction or interruption in the early phase, while the low‐dose group managed to extend the period of dose reduction or interruption with some patients, even those undergoing dose escalation. It became evident after 6 weeks that both groups had reached median average daily dose of approximately 20 mg. Although there were no significant differences in PFS and OS between the two groups, median PFS and OS in the low‐dose group were 4.0 and 16.7 months. Conversely, patients who received a 6 W‐RDI≥33.4% of cabozantinib exhibited a tendency toward a longer median PFS compared with that of those who received <33.4%. We also clarified that the serum aldolase value could be a surrogate marker of cabozantinib exposure and a value of 8.7 U/L or more was associated with a very early dose reduction or interruption.

In the current study, 89.1% in all patients experienced dose reduction or interruption due to AEs and the median average daily dose of cabozantinib was 20 mg during the first 6 weeks. According to the Japanese phase 2 trial for patients with uHCC treated with cabozantinib, the dose reduction and interruption occurred in 91% and 91%, respectively, and the median daily dose of cabozantinib was 22 mg.^4^ Our cabozantinib administration profiles are relatively consistent with those of the trial. In contrast, the international phase 3 trial for HCC, which did not include Japanese patients, reported a 62% dose reduction, 84% dose interruption, and median daily dose of 36 mg of cabozantinib,^3^ indicating that the administration profiles of cabozantinib differ from those in Japanese patients.

Similarly, the median daily dose of 26 mg cabozantinib in the Japanese phase 2 trial for renal cell cancer was shown to be lower than that of 43 mg cabozantinib in the international phase 3 trial.^10^, ^11^ Moreover, cabozantinib exposure in Japanese patients with non‐small cell lung cancer have been reported to be 30% higher than those in non‐Japanese patients.^12^ Based on these reports including the examination of other cancers and the present result, an initial dose of 60 mg has the potential for overexposure, especially in Japanese patients. Although one reason for the increased exposure in Japanese patients compared with European or American patients is considered to be a body size difference,^13^ the other reasons including any pharmacological factor might be associated with the ethnic differences between Japanese and the other race.

Real‐world data from Japan suggest that an initial dose of either 40 or 20 mg cabozantinib is commonly prescribed to patients with HCC due to its pharmacological properties.^5^, ^6^, ^7^, ^8^ Among these, Tomonari et al.^5^ demonstrated the feasibility of a reduced‐dose group, starting at 40 or 20 mg, compared with the full‐dose group in a cohort of 26 patients. Conversely, the present study investigated the efficacy and toxicity between the 20 and 40 mg or more groups. To the best of our knowledge, this is the first study to compare the clinical outcomes in the 20 mg group with those in the 40 or 60 mg groups. As expected, we clearly showed that the duration of the first dose interruption in the 20 mg dose was significantly prolonged compared with 40 mg or more due to the prevention of common AEs. In summary, starting with 20 mg cabozantinib is a safe treatment option for avoiding early drug interruption.

Recent data including the use of cabozantinib after immunotherapy regimens have shown that the median PFS was between 2.1–5.1 months, and OS was 4.3–12.1 months.^6^, ^7^, ^8^, ^14^, ^15^, ^16^, ^17^ The clinical outcomes of the current cohort that median PFS and OS in all patients was 4.0 and 11.6 months are consistent with these past reports. In addition, the therapeutic efficacy in the low‐dose group that median PFS and OS were 4.0 and 16.7 months were satisfactory compared to the previous reports. Taken together, 20 mg of cabozantinib could be an acceptable initial dose for Japanese patients.

The present study shows the safety of the initial cabozantinib dose of 20 mg. Nevertheless, it remains uncertain whether the treatment efficacy was superior at the lower or higher starting doses due to the difference in baseline AFP value. Notably, the present results focusing on the RDI indicated that there were no significant differences in the 6 W‐RDI regardless of whether high‐ or low‐starting dose were administered and that patients with a 6 W‐RDI≥33.4% tend to have improved PFS compared with those of <33.4%. Despite the ongoing administration of 20 mg cabozantinib for the first 6 weeks, the 6 W‐RDI only attains 33.3%. Notably, dose escalation was required for 27.8% of the low‐dose group. Consequently, it is essential to determine the appropriate timing for increasing the cabozantinib dose, particularly in the absence of AEs with the initial dose of 20 mg. Based on the result shown in Table 6, it is reasonable to consider dose escalation at 2 weeks in the case of aldolase <8.7 U/L.

Recent studies have demonstrated substantial variability in cabozantinib clearance and exposure among individuals. Additionally, there is considerable inter‐individual variability in cabozantinib clearance and exposure.^9^, ^18^ Moreover, the half‐life of cabozantinib is 120 h, indicating a feature of high accumulation among various TKI.^19^ Our results that dose reduction or interruption rate during 6 weeks was 79.7% even in the low‐dose group imply the difficulty in initial dose setting and dose adjustment during cabozantinib treatment. The pharmacokinetic‐pharmacodynamic analysis of cabozantinib for HCC has shown that its efficacy and safety are dependent on the level of exposure.^20^ In a previous study, we reported preliminary data indicating that the C_trough_ of cabozantinib was associated with changes in serum aldolase and CK levels from baseline in a cohort of 14 Japanese patients with uHCC.^9^

In the present study, we investigated the updated data on clinical efficacy and toxicity in a cohort of 34 patients. We also examined the relationship between drug exposure and serum markers, including aspartate aminotransferase and LDH, which would reflect indirect muscle injury in 18 patients. As expected, not only aldolase and CKvalues, but also aspartate aminotransferase and LDH were related to the C_trough_ of cabozantinib. These results indicate that muscle injury is closely associated with cabozantinib exposure. Aldolase, a glycolytic enzyme that is widely distributed in the living body, is often measured along with serum CK in cases of muscle injury.^21^ Serum aldolase, rather than CK and LDH, is also considered to be a more sensitive marker for exercise‐induced muscle damage.^22^ A previous study reported that severe anorexia developed in patients with high exposure to lenvatinib for HCC.^23^ Similarly, with regard to cabozantinib treatment, it is clinically meanigful that aldolase levels, which could be related to cabozantinib exposure, were associated with the severity of anorexia in the current study. In addition, a recent retrospective study showed that CK elevation occurred in 61.5% of patients during cabozantinib treatment for advanced renal cell carcinoma.^24^

The precise mechanism of cabozantinib‐induced muscle injury requires further clarification. In general, tyrosine kinase receptors are considered to activate the phosphoinositide 3‐kinase (PI3K)/thymoma viral proto‐oncogene (AKT)/mechanistic target of rapamycin kinase (mTOR) pathway, an intracellular signaling pathway for protein synthesis and proteolysis. A recent in vitro and animal study clarified that lenvatinib, a TKI, induces muscle injury by decreasing mitochondria‐related proteins in skeletal muscle.^25^ Similarly, cabozantinib might evoke muscle injury by suppressing PI3K/AKT/mTOR signaling. Another potential mechanism is that cabozantinib inhibits Fms‐like tyrosine kinase 3 (FLT‐3), which is a tyrosine kinase receptor primarily found on the membrane of hematopoietic progenitor cells.^26^ Additionally, the FLT‐3 ligand and FLT‐3 signaling pathway are expressed in differentiating myoblasts and their signaling plays a vital role in the regulation of skeletal myogenesis.^27^

Dose reduction or interruption of cabozantinib at the very early stage, within 2 weeks after starting treatment, could be regarded as an error in the initial dose setting. We clearly showed that the serum aldolase value was associated with dose reduction or interruption at a very early stage in the multivariate analysis. Although CK and aldolase elevations are asymptomatic and might not be clinically problematic, the novel finding that cabozantinib induces asymptomatic muscle injury is of utmost importance in clinical management, and clinicians should be aware that these elevations are highly suggestive of dose elevation of cabozantinib. In particular, the baseline value of aldolase in our cohort, with a coefficient of variation of 20.1% (data not shown), could contribute to the acceptable clinical use of aldolase values. Cabozantinib treatment is often chosen as a later line of uHCC therapy, with the objective of sustaining stable disease. To accomplish this, it is crucial to avoid prolonged interruptions or cessation of the drug. Concerning dose reduction for grade 2 AEs, aldolase levels would determine the need for dose reduction or intensive monitoring. To the best of our knowledge, this is the first report to identify surrogate markers for AE management in patients with uHCC treated with cabozantinib.

Our study has several limitations. First, the study was retrospective nature of the analysis and had a potential selection bias. Specifically, the early realization in attending physicians that an initial cabozantinib dosage of 60 mg leads to early dose reduction or interruption could influence the decision of the starting dose. Second, regarding the comparison of therapeutic outcomes between the high‐dose and low‐dose groups, caution is required because the baseline AFP value was significantly higher in the high‐dose group. Third, the study included two institutions in a comparatively limited number of patients. To obtain concrete results of an adequate initial dose of cabozantinib, a larger prospective comparative study would be needed. However, the serum aldolase values were examined with a routine and novel findings related to aldolase values were found, because the current study was conducted at two related institutions within the same university. Fourth, a decision on cabozantinib dose modification, especially in non‐acceptable grade 2 AE, was according to the discretion of attending physicians, also leading to bias. Fifth, as the current study population comprised Japanese individuals, the validity of an initial dose of 20 mg of cabozantinib can only be determined in Japanese patients. Further research is required to determine the optimal initial dose of cabozantinib for different ethnic groups. Despite these limitations, the real‐world clinical data, including the dose setting and modification of cabozantinib, may be useful in improving the therapeutic efficacy and reducing treatment‐related adverse effects in patients with uHCC undergoing cabozantinib treatment.

In conclusion, our findings suggest that an initial dose of 20 mg cabozantinib is a safe option in Japanese patients and it is advisable to consider the dose escalation. The serum aldolase level, which serves as a reliable indicator of cabozantinib exposure, may be useful for making appropriate dose modifications.

## AUTHOR CONTRIBUTIONS

**Hironao Okubo:** Conceptualization (lead); data curation (lead); formal analysis (lead); investigation (lead); methodology (lead); project administration (lead); resources (lead); validation (lead); writing – original draft (lead); writing – review and editing (lead). **Hitoshi Ando:** Formal analysis (supporting); investigation (supporting); methodology (supporting); project administration (supporting); writing – review and editing (supporting). **Shunsuke Nakamura:** Data curation (supporting). **Yusuke Takasaki:** Data curation (supporting). **Koichi Ito:** Data curation (supporting). **Yuka Fukuo:** Data curation (supporting). **Kenichi Ikejima:** Formal analysis (supporting); supervision (equal). **Hiroyuki Isayama:** Supervision (equal); writing – review and editing (supporting).

## CONFLICT OF INTEREST STATEMENT

Authors declare no Conflict of Interests for this article.

## ETHICS STATEMENT

The study was approved by the Juntendo University School of Medicine Ethics Committee for Medical Research (Approval No. E23‐0193) and was performed in accordance with the ethical standards of the 1964 Declaration of Helsinki and its later amendments.

## PATIENT CONSENT STATEMENT

Informed consent was obtained from all the patients to participate in the study.

## Supporting information


**Figure S1.** The time course changes in serum aldolase, creatine kinase, aspartate aminotransferase, alanine aminotransferase, and lactic acid dehydrogenase levels in all patients.

## Data Availability

The dataset that support the findings of this study are available from the corresponding author upon reasonable request.
