# Supplementary figures and images for: Real world data of cabozantinib in patients with hepatocellular carcinoma: Focusing on dose setting and modification
Source: Cancer Med. 2024 Sep 24;13(18):e70222. doi: 10.1002/cam4.70222 (PMC11420626; doi:10.1002/cam4.70222)

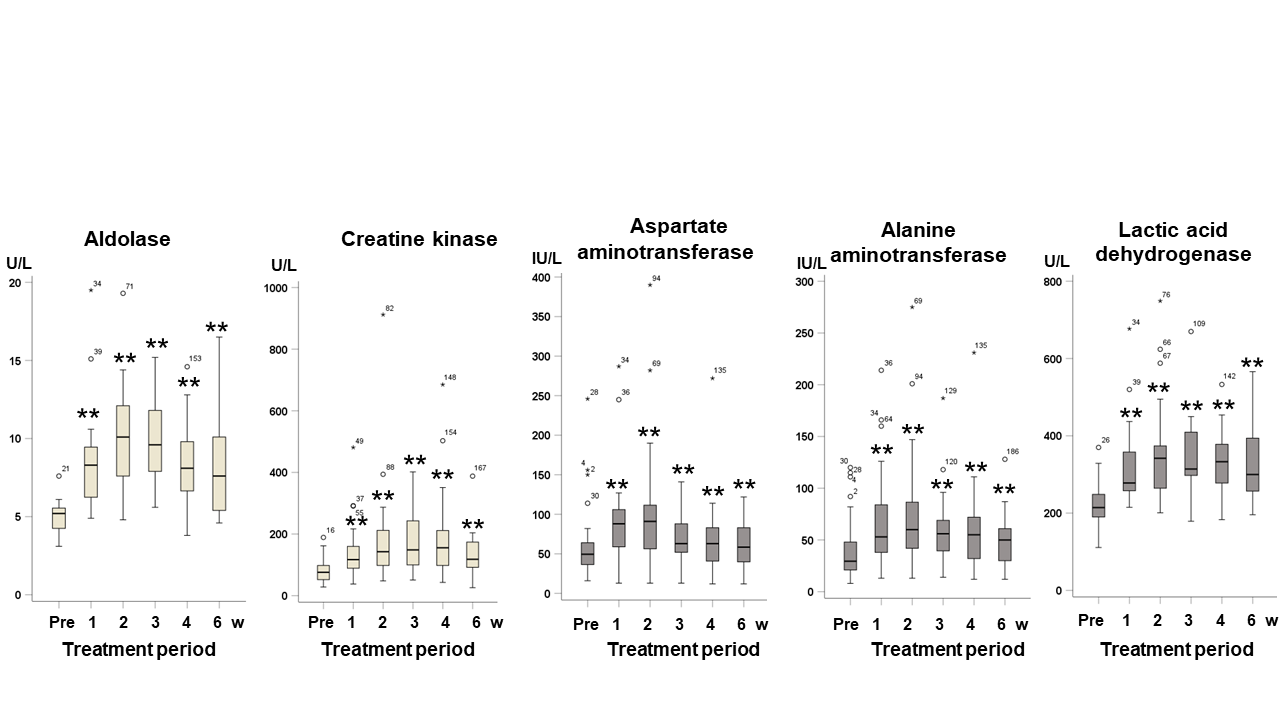

Supplement: Supplementary file 1 — Figure S1. The time course changes in serum aldolase, creatine kinase, aspartate aminotransferase, alanine aminotransferase, and lactic acid dehydrogenase levels in all patients. [file CAM4-13-e70222-s001.tif]
